# Screening for γ-Nonalactone in the Headspace of Freshly Cooked Non-Scented Rice Using SPME/GC-O and SPME/GC-MS

**DOI:** 10.3390/molecules14082927

**Published:** 2009-08-10

**Authors:** Zhi Zeng, Han Zhang, Tao Zhang, Shigeru Tamogami, Jie Yu Chen

**Affiliations:** 1 School of Chemistry and Environment, South China Normal University, Guangzhou 510631, China; E-mail: zhizeng@scnu.edu.cn (Z.Z.); 2 Faculty of Bioresource Sciences, Akita Prefectural University, Kaidobata-Nishi 241-438, Shimoshinjo-Nakano, Akita-shi, Akita 010-0195, Japan; 3 Institute of Opto-electronic Materials and Technology, South China Normal University, Guangzhou 510631, China

**Keywords:** rice (*Oryza sativa* L.), aroma, γ-nonalactone, gas chromatography-olfactometry (GC-O), gas chromatography-mass spectrometry (GC-MS)

## Abstract

The determination of γ-nonalactone as one of the important odor-active compounds in freshly cooked non-scented rice is reported. It was evaluated by gas chromatography-olfactometry (GC-O) analysis and identified by gas chromatography-mass spectrometry (GC-MS) analysis in the headspace above the freshly cooked non-scented rice samples extracted by using a modified headspace solid-phase microextraction (SPME) method. This component had a mass spectrum with a characteristic ion peak at *m/z* 85 (100%) and a linear retention index (RI) of 2,023 on a DB Wax column, consistent with those of an authentic sample of γ-nonalactone. The odor characterization of a strong, sweet, coconut-like aroma of this compound was also validated by GC-O comparison with the authentic compound.

## 1. Introduction

A considerable number of different rice (*Oryza sativa* L.) cultivars are grown throughout the World. Japanese consumers seem to prefer the more bland non-scented cultivars, whereas in South East Asia, India, and Middle Eastern countries a number of scented cultivars of rice are highly favored.

Determination of volatile compounds present in cooked samples of various rice cultivars has been the topic of a number of investigations, and more than 100 volatiles have been reported [1,2]. However, only a few compounds have been found to have an impact on the aroma of cooked rice [3,4,5,6,7,8]. In scented rice, the strong popcorn-like smell of 2-acetyl-1-pyrroline was identified by Buttery *et al*. [5,6]. Jezussek *et al*. [9] determined the odorants in cooked brown rice of four cultivars using aroma extract dilution analysis (AEDA). More recent work by Yang *et al*. [10] reported that 35 volatile compounds were identified by gas chromatography-mass spectrometry (GC-MS) using a dynamic headspace system with Tenax trapping in cooked black rice, and 25 compounds emanating from black rice were characterized as odor-active based upon gas chromatography-olfactometry (GC-O).

Solid-phase microextraction (SPME) is an interesting and promising technique for the extraction and concentration or enrichment of volatile compounds from different sample matrices [11,12,13,14]. It is a growing sample preparation technique, and an attractive alternative to conventional extraction methods, that reduces solvent usage and exposure, disposal costs and extraction time for sample separation and concentration purposes. This technique has been used in studies on the flavor indicators of rice [4,15,16]. Champagne *et al*. [17,18] investigated the volatile microbial metabolites gathered in rice based on the SPME technique. 

In our previous studies, a modified headspace SPME method was developed for the analysis of flavor volatiles of rice and a number of volatile components have been identified in the headspace of rice during cooking [19]. By using this method, the flavor volatiles of freshly cooked rice could be determined [20]. However, the chemical definition of the aroma of non-scented rice has not been adequately achieved. This study was undertaken to determine the strong, sweet, coconut-like odorant in freshly cooked non-scented rice samples using the modified headspace SPME method coupled with GC-O and GC-MS. Comparisons between the spectra of GC-O and GC-MS of the flavor volatiles in the headspace above the freshly cooked rice samples were used to determine which component was associated with a strong, sweet, coconut-like aroma.

## 2. Results and Discussion

### 2.1. Characteristic sweet, coconut-like aroma component of non-scented rice

**Figure 1:**
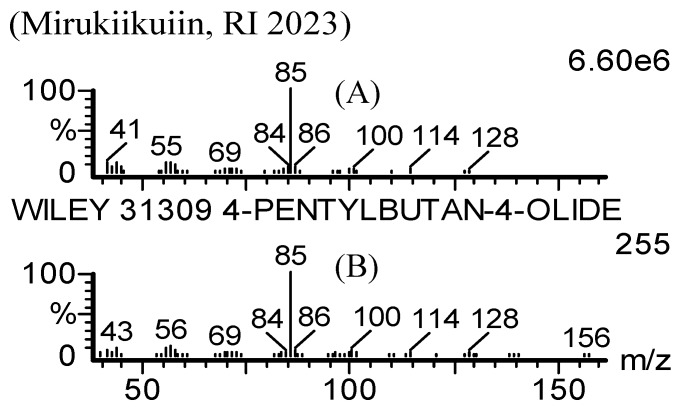
Mass spectrum observed for the chromatographic peak at an RI of 2023 on a DB Wax column with a strong, sweet, coconut-like aroma in the headspace above freshly cooked Mirukiikuiin rice (**A**) and mass spectrum of γ-nonalactone (also known as 4-pentyl-butan-4-olide) in the Wiley mass spectral library (31309) (**B**).

The flavor volatiles were extracted from the freshly cooked rice samples using a modified headspace SPME sampling with the headspace inside an automatic rice cooker instead of in a vial, as described in detail in the Experimental. Modified headspace SPME coupled with GC-O and GC-MS analyses were carried out on the odorants from the flavor volatiles of freshly cooked Mirukiikuiin rice samples. One of the prominent peaks, with an RI of 2,023 on the DB Wax column and with a strong, sweet, coconut-like aroma was found in the headspace above the freshly cooked rice. This component showed a mass spectrum with a characteristic ion peak at *m/z* 85 (100%), as illustrated in Figure 1. The RI and mass spectrum data were consistent with those for an authentic sample of γ-nonalactone, and the odor characterization of a strong, sweet, coconut-like aroma was also validated by GC-O with the authentic compound. The presence of this compound was further confirmed by modified headspace SPME coupled with GC-O and GC-MS analyses of the flavor volatiles from other freshly cooked non-scented rice samples, such as LGC-soft and LGC-katsu.

γ-Nonalactone is related to a group of “sweet, coconut-like aroma” compounds which includes several γ-lactones such as γ-octalactone, γ-decalactone, and γ-dodecalactone. With an odor threshold of 30 ppb in water [21], this component appears to be one of the important aromas of this group.

### 2.2. Determination of the amount of γ-nonalactone in freshly cooked rice samples

Unlike environmental samples, where the target compounds are typically present in trace amounts, analyte concentrations in food samples are variable and dependent on the analytical conditions employed at their extraction and concentration [11,13]. This is particularly true for compounds found at trace levels. Hence, reports of the amounts of trace levels of endogenous analytes in foods must present all of the experimental details, because meaningful comparisons can be made only between experiments that subject the sample to similar conditions [4]. On the other hand, the sample must reach a temperature sufficient either to liberate bound volatile compounds or to thermally generate the compounds, in order to successfully sample the headspace above freshly cooked rice with modified headspace SPME. The temperature variation in the headspace above the freshly cooked rice sample was reported previously as that at the stage of keeping the cooked rice warm for 30 min [19]. All of the free flavor volatiles, the bound flavor components and the thermally generated compounds could be liberated during rice cooking and then extracted, detected and identified in the headspace above freshly cooked rice at the stage of keeping the cooked rice warm. For this reason, the modified headspace SPME method for the study of the aroma of freshly cooked rice is preferred to other sampling techniques. Determination of the amount of γ-nonalactone in freshly cooked rice samples was carried out using chromatographic peak area counts in total ion current (TIC) chromatograms and no external or internal standards were used [22]. Using peak area counts for quantity comparison does not require extensive sample preparation, but the sampling procedure and chromatographic conditions must remain constant for all samples. Table 1 compares the relative amounts (chromatographic peak area counts × 10^6^) of γ-nonalactone found in the headspace above four freshly cooked rice samples, which were tested under the same operating conditions. The chromatographic peak areas shown are the means of three determinations with standard deviations. The data in Table 1 are only intended to give some idea of the order of magnitude of the amounts. To obtain more reliable results, a calibration method should be used.

**Table 1:** Relative amount and odor intensity of γ-nonalactone in the headspace above the freshly cooked rice samples of three non-scented and one scented cultivars.

Rice cultivar	Relative amount ^a^	Odor intensity ^b^
Non-scented rice		
LGC-soft	13.72±1.63	5.0±0.0
Mirukiikuiin	8.14±0.46	5.0±0.0
LGC-katsu	4.63±0.22	4.7±0.5
Scented rice		
Hieri	nd ^c^	nd

^a^ Relative amount of γ-nonalactone was in TIC chromatographic peak area (area counts × 10^6^) [22]. Results expressed as mean ± standard deviation (*n* = 3); ^b^ Odor intensity values of the sweet, coconut-like aroma for the chromatographic peak at an RI of 2023 on a DB Wax column were averaged for six runs (three panelists in duplicate, 3 × 2) by GC-O (posterior intensity method) [23]. Results expressed as mean ± standard deviation (*n* = 6); ^c^ Not detected.

Of all components identified in the headspace above the freshly cooked rice samples by modified headspace SPME coupled with GC-O and GC-MS (data not shown), γ-nonalactone was among the components with the most average intensity of aroma for Mirukiikuiin, LGC-soft and LGC-katsu, as presented in Table 1. This component can be one of the important odor-active compounds in freshly cooked rice for the three cultivars of non-scented rice. However, this component was not detected in Hieri, a scented rice cultivar (Table 1).

In previous studies carried out on rice aroma, γ-nonalactone was not reported as one of the important odorants [7,8,10]. Jezussek *et al*. [9] only listed it as one of the minor odor-active compounds in a cooked “Indica” brown rice, with the flavor dilution (FD) factor of 1, maybe because of its low concentration in the rice samples. Moreover, this component was found as one of the flavor volatiles in the cooked rice of a scented Kaorimai cultivar [24]. It was recently reported as one of the volatiles in non-scented rice types such as Nihonbare, Koshihikari and Akitakomachi by our group [20]. Besides, γ-nonalactone was identified as one of the volatile components in the steam distillate of rice bran [25,26] and one of the constituents of volatile oils from rice straw [27]. Although not previously considered in cooked rice, γ-nonalactone has been suggested as one of the important odor-active compounds in various foods, such as fresh wheat germ [28], as well as in various wines [21,29,30,31]. 

## 3. Experimental

### 3.1. Rice samples and authentic compound

Samples of one scented and three non-scented cultivars of rice were obtained. The non-scented ones, Mirukiikuiin, LGC-soft and LGC-katsu were harvested in Akita Prefecture, Japan, while the scented kind, Hieri was harvested in Kouchi Prefecture, Japan. They were dehulled at the growing area and were transported to the laboratory in the form of brown rice, and then stored at 4 ºC until experiments were performed. The rice samples were milled by an RSD-A100 milling machine (TIGER, Japan) to a 90% milling yield (brown rice basis) to give white rice, which were used for the experiments. An authentic sample of γ-nonalactone was purchased from Wako Pure Chemical Industries, Japan. This substance was dissolved in acetone in a concentration of 1% (v/v), and the solution (1 µL) was subjected to GC-O and GC-MS analyses.

### 3.2. Headspace solid-phase microextraction sampling

The traditional Japanese rice cooking method was used, by which a mixture of white rice (150 g) and distilled water (250 mL) was placed in an automatic electric rice cooker (SR-A18H, National, Japan). The mixture was heated for 48 min until the automatic stop of heating and kept warm for another 30 min in order to cook completely. A thermo-recorder (TR-72S, T & D, Japan) was used for the measurement of the variation in temperature in the headspace above the freshly cooked rice sample.

Extraction and concentration of the flavor volatiles of freshly cooked rice were performed using an SPME fiber (Supelco) 1 cm long, coated with triple-phase 30/50 μm divinylbenzene/carboxen/poly-dimethylsiloxane (DVB/CAR/PDMS), preconditioned in an SPME fiber conditioner (GL Sciences) at 250 **º**C for 1 hr before the first measurement. While keeping the cooked rice warm for 30 min, the SPME fiber, mounted in the manually operated SPME holder, was in its protective sheath when it was inserted directly into the automatic electric rice cooker through a Teflon-coated silicone septum that tightly covered the open part of the cooker after automatic stop of heating. Inside the cooker, it was exposed to the headspace above the freshly cooked rice sample and left there for adsorption for 30 min. The SPME fiber thermally desorbed the flavor volatiles in the injection port of the GC-O or GC-MS instruments for 5 min at 250 ºC. Then it was left in the SPME fiber conditioner at 250 ºC for 1 h for reconditioning before it was exposed to the headspace of the next sample. The DVB/CAR/PDMS fiber was used in all applications.

### 3.3. Gas chromatography-olfactometry

Gas chromatography-olfactometry (GC-O) is comprised of a GC-2010 gas chromatograph (Shimadzu, Japan) equipped with a flame ionization detector (FID, Shimadzu) and an olfactory detector outlet (ODO II control module, SGE, Japan) with a glass nose cone. The GC-2010 was equipped with a split/splitless injector. Desorption time was 5 min in the splitless mode in the injection port at 250 ºC. A column, DB Wax, 30 m × 0.25 mm i.d. × 0.25 µm (stationary phase thickness) (J & W Scientific) was applied. It was temperature-programmed at 40 ºC for 2 min, then increased to 230 ºC at a rate of 4 ºC min^-1^, and maintained at 230 ºC for 4.5 min. The carrier gas was helium, and the column head pressure was 114.6 kPa at a constant linear velocity of 35 cm sec^-1^. The FID temperature was 250 ºC. The following gases and flow rates were used for the FID system: the makeup gas was N_2_ at a flow rate of 50 mL min^-1^; the H_2_ flow rate was 50 mL min^-1^; the air flow rate was 400 mL min^-1^. Data were collected by GC Solution software (Shimadzu).

At the end of the DB Wax capillary column, the gas flow was split into two equal parts according to the small amount injected through SPME fiber: one part going to the FID and the other going to the sniffing cone. The split occurred through an SGE column flow splitter connected to two deactivated and uncoated fused silica capillaries as transfer-line tubing of the same length (1.10 m × 0.25 mm i.d.). Auxiliary gas for ODO II was helium at 3.5 on the flow-meter scale (ca. 12 mL min^-1^). The sniffing cone was purged with humidified air to help maintain olfactory sensitivity by reducing dehydration of the mucous membrane in the nasal cavity. Condensation of the effluents was avoided by heating the transfer-line tubing.

GC-O analyses were carried out on the odorants of each freshly cooked rice sample after modified headspace SPME sampling. Trained panelists were asked to characterize detectable odors with a description and an indication of intensity on a scale ranging from 1 to 5: (1, very weak; 2, weak; 3, moderate; 4, strong; 5, very strong) [23]. Each odorant was screened by three trained panelists (one male and two females) by sniffing the effluent after gas chromatographic separation. Sniffing analyses were repeated twice by each panelist. The retention time, odor intensity and odor description were recorded. The retention time of each odorant was converted to the linear retention index (RI) using *n*-alkanes (Supelco) as the references. The intensity values were averaged for the three panelists in duplicate (3 × 2).

### 3.4. Gas chromatography-mass spectrometry

Gas chromatography-mass spectrometry (GC-MS) analyses were conducted on a TurboMass GC mass spectrometer with AutoSystem XL and TurboMass Upgrade MS software (Perkin Elmer). Desorption time was 5 min in the injection port at 250 ºC, with a split ratio of 5:1. The same column, injection conditions and oven temperature programming as for GC-O analyses were used. The carrier gas was helium, which was delivered at a linear velocity of 2 mL min^-1^. The mass selective detector was operated in an electron impact ionization mode at 70 eV, in a scan range of *m/z* 40–400. The interface temperature was 230 ºC. Retention time of each volatile was converted to the linear retention index (RI) using *n*-alkanes (Supelco) as the references. Each volatile compound was positively identified by matching its mass spectrum and RI value with those of an authentic sample. The results from the volatile analyses were provided in chromatographic peak area counts [22]. All experiments were performed in triplicate.

## 4. Conclusions

Through application of the methodology of modified headspace SPME coupled with GC-O and GC-MS, γ-nonalactone has been determined as one of the important odor-active compounds in freshly cooked non-scented rice, such as Mirukiikuiin, LGC-soft and LGC-katsu. Although more quantitative work is needed to confirm the present results, this preliminary finding will help to further understand the aroma and flavor in freshly cooked non-scented rice.
